# Mesenchymal dental stem cells in regenerative dentistry

**DOI:** 10.4317/medoral.17925

**Published:** 2012-08-28

**Authors:** Francisco-Javier Rodríguez-Lozano, Carmen-Luisa Insausti, Francisca Iniesta, Miguel Blanquer, María-del-Carmen Ramírez, Luis Meseguer, Ana-Belén Meseguer-Henarejos, Noemí Marín, Salvador Martínez, José-María Moraleda

**Affiliations:** 1 Cell Therapy Unit. Hospital Universitary Virgen de la Arrixaca, Faculty of Medicine and Odontology, University of Murcia, Spain; 2 Institute of Neurosciences. University Miguel Hernández. Alicante. Spain

## Abstract

In the last decade, tissue engineering is a field that has been suffering an enormous expansion in the regenerative medicine and dentistry. The use of cells as mesenchymal dental stem cells of easy access for dentist and oral surgeon, immunosuppressive properties, high proliferation and capacity to differentiate into odontoblasts, cementoblasts, osteoblasts and other cells implicated in the teeth, suppose a good perspective of future in the clinical dentistry. However, is necessary advance in the known of growth factors and signalling molecules implicated in tooth development and regeneration of different structures of teeth. Furthermore, these cells need a fabulous scaffold that facility their integration, differentiation, matrix synthesis and promote multiple specific interactions between cells. In this review, we give a brief description of tooth development and anatomy, definition and classification of stem cells, with special attention of mesenchymal stem cells, commonly used in the cellular therapy for their trasdifferentiation ability, non ethical problems and acceptable results in preliminary clinical trials. In terms of tissue engineering, we provide an overview of different types of mesenchymal stem cells that have been isolated from teeth, including dental pulp stem cells (DPSCs), stem cells from human exfoliated deciduous teeth (SHEDs), periodontal ligament stem cells (PDLSCs), dental follicle progenitor stem cells (DFPCs), and stem cells from apical papilla (SCAPs), growth factors implicated in regeneration teeth and types of scaffolds for dental tissue regeneration.

** Key words:**Dental stem cells, regenerative dentistry, mesenchymal stem cells, tissue engineering, stem cells.

## Introduction

The formation of the tooth is determined by the cells of which it is composed, the buccal epithelial cells that form the enamel organ and the mesenchymal cells that form the dental papilla. The enamel is formed by the enamel organ, and the dentin is formed by the dental papilla. Cells from the neural crest also take part in tooth formation. These cells originate in the nervous system and later migrate to the maxilla and mandible, where they interact with mesenchymal cells to form the enamel organ and the dental papilla (1,2).

**Figure 1:**
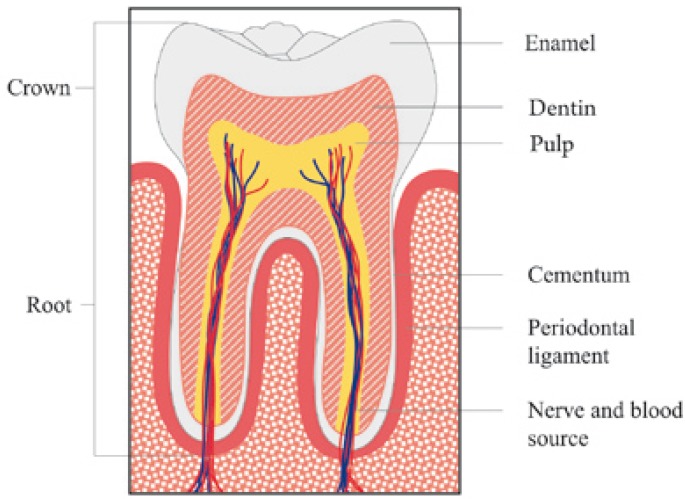
Tooth anatomy.

The tooth has two anatomical parts. The crown is the part of the tooth which is covered with enamel and it is the part usually visible in the mouth. The root is the part embedded in the jaw. It anchors the tooth in its bony socket and is normally not visible. The tissues of tooth are enamel, dentin, cementum and pulp. The pulp contains blood vessels and nerves that enter the tooth from a hole at the apex of the root and cementum (Fig.1). Around of tooth the periodontal ligament attaches the cementum to the alveolar (3).

-Dental Pulp Tissue

Dental pulp is a loose connective tissue that occupies the pulp chamber of the tooth and originates in the embryonic dental papilla (ectomesenchymal tissue); it is the mature form of the papilla and the only smooth tissue of the tooth. The principal cell of this tissue is the odontoblast, also referred to as the dentinoblast. The dental pulp also contains fibroblasts, undifferentiated mesenchymal cells or stem cells, macrophages, and lymphocytes (4).

-Periodontal ligament (PDL)

The periodontal ligament (PDL) is a vascularised, cellular soft connective tissue that surrounds the teeth and joins the root cementum with the hard sheet of the alveolar bone (5). Most of the cells in the PDL are fibroblasts, which primarily function to synthesise and maintain the extracellular matrix. These fibroblasts contain a developed cytoskeleton of microtubules and actin microfilaments that has been implicated in cellular motility processes. In addition to fibroblasts, the PDL contains osteoblasts, osteoclasts, cementoblasts, macrophages, and stem cells that are capable of generating fibroblasts, cementoblasts, and osteoblasts (6,7).

## Stem Cells

The term stem cell was proposed for scientific use by Russian histologist Alexander Maksimov in 1909. Alexander Maximov was the first to suggest the existence of hematopoietic stem cells (HSC) with the morphological appearance of a lymphocyte, capable of migrating throughout the blood to microecological niches that would allow them to proliferate and differentiate along specific (8). While research on stem cells grew out of findings by Canadian scientists in the 1960s (8,9). Based on their origin, there are two main types of stem cells: embryonic stem cells (ES cells) and postnatal or adult stem cells (AS cells). Embryonic stem cells were harvested from embryos, they are cells derived from the inner cell mass of the blastocyst (early stage embryo, 4-5 days old, consist of 50-150 cells) of earlier morula stage embryo. In other words these are the cells that form the three germ layers, and are capable of developing more than 200 cell types. In 1998 the first human embryonic stem cell line was derived at university of Wisconsin-Madison (10).

Stem cells can be classified according to their abilities to differentiate as totipotent, pluripotent, or multipotent. Totipotent stem cells are those that can be implanted in the uterus of a living animal and give rise to a full organism. Pluripotent stem cells are those that can give rise to every cell of an organism except its extra-embryonic tissues, such as the placenta. This limitation re-stricts pluripotent stem cells from developing into a full organism. Embryonic stem (ES) cells and induced pluripotent stem (iPS) cells are pluripotent stem cells. Multipotent stem cells are adult stem cells which only generate specific lineages of cells (11,12).

Embryonic stem cells have both moral and technical problems; because these cells will later develop into a human being, taking these cells will require destruction of an embryo. Technically these cells are difficult to control and grow and they might as well form tumors after their injection (13). Differentiating embryonic stem cells into usable cells while avoiding transplant rejection are just a few of the hurdles that embryonic stem cell researchers still face. And after ten years of research, there are no approved treatments or human trials using embryonic stem cells; but because of the combined abilities of unlimited expansion and pluripotency, embryonic stem cells remain a theoretically potential source of regenerative medicine and tissue replacement after injury or disease (14).

A very recent development, with potentially a profound significance for clinical therapy has been the generation of induced pluri-potent stem (iPS) cells from somatic cells. The method for iPS cell induction is “ground-breaking” because somatic cells are converted directly into pluripotent cells through introduction of four genes: Oct-4, Sox2, c-Myc and Klf4 (15). iPS cells have been shown to be similar to ES cells in morphology, proliferation and differentiation capacity and genomic and epigenomic states (16).

To date, AS cells provide a promising tool for clinical applications in the near future due to their accessibility, despite their reduced plasticity (11). Although limited in their capability to differentiate, they can still develop into a number of cell lineages. The possibility of harvesting postnatal stem cells for later use in the same patient eliminates immunological difficulties and the risk of pathogen transmission. Adult stem cells from autologous origin are an appealing, and practical source for cell-based regenerative therapies that hold realistic clinical potential (11).

-Mesenchymal Stem Cells

Alexander Friedenstein was the first to evidence the presence of a population of nonhematopoietic cells that were capable of autorenovation and bone differentiation in the bone marrow (17). Subsequently, others showed the bone-marrow-derived cells isolated according to Friedenstein’s technique, also possessed high potency of proliferation and pluripotency of differentiation into mesenchymal tissues, and therefore Caplan used the term ‘‘mesenchymal stem cell’’ (MSC) to describe them (18). Further studies have established mesenchymal stem cells as a heterogeneous cell population in which each individual cell varies in its gene expression, differentiative capacity, expansion potential and phenotype (19,20). Moreover, all of them do not seem to fulfill the stem cell criteria. Therefore, they are preferred to be called ‘‘multipotent stromal cell’’ with the same acronym “MSC” (20). Several studies have demonstrated that MSCs can be isolated from multiple tissues, such as bone marrow, peripheral blood, umbilical cord blood, adult connective tissue, dental tissues, placenta, and amniotic membrane (21-24).

At present, any cell population which meets the following characteristics, irrespective of its tissue source, is generally referred as MSC: morphologically, they adhere to plastic and have a fibroblast-like appearance; functionally, they have the ability of self-renewal and could differentiate into cells of the mesenchymal lineage (osteocyte, chondrocyte and adipocyte), also into cells of the endoderm (hepatocytes) and ectoderm (neurons) lineages under proper cell culture conditions; phenotypically, they express more than 95% of the population express the CD105, CD73,CD90 surface antigens and that less than 2% of the population ex-press the pan-leukocyte marker CD45, the primitive hematopoietic progenitor and endothelial cell marker CD34, the monocyte and macrophage markers CD14 and CD11, the B cell markers CD79 and CD19, or HLA class II (25).

-Tissue Engineering in Dentistry with Mesenchymal dental stem cells

Tissue engineering is an interdisciplinary field of study that applies the principles of engineering to biology and medicine toward the development of biological substitutes that restore, maintain, and improve normal function (26). The emerging discipline of tissue engineering and regenerative medicine endeavors to use a rational approach based on morphogenetic signals for tissue induction, responding stem/progenitor cells and the scaffold to maintain and preserve the microenvironment (26).

Growth factors

Growth factors and signaling molecules have the ability to stimulate cellular proliferation and cellular differentiation. Bone morphogenetic proteins (BMPs) family members are used sequentially and repeatedly throughout embryonic tooth development, initiation, morphogenesis, cytodifferentiation and matrix secretion (26). Six different Bmps (Bmp2–Bmp7) are coexpressed tem-porally and spatially Bmp6 were identified in human primary culture of dental pulp cells (27). BMPs have been successfully ap-plied for the regeneration of periodontal tissue (28), and other factors, such as PDGF, IGF-1, FGF-2, TGF-β, and BMPs (29), have utility in tooth tissue engineering. Dentin matrix protein-1, a non-collagenous protein involved in the mineralization process, induced cytodifferentiation, collagen production and calcified deposits in dental pulp in a rat model (27). Other investigations have been demonstrated the effect of dexamethasone in cultures with dental stem cells, where these cells in combination with dexa-methasone can differentiate into osteoblasts, adipocytes or chondrocytes (30). Recently has been comproved the role of 17β-estradiol on cementoblast activity. An in vitro study with PDL fibroblasts showed enhanced alkaline phosphatase activity and mineralized nodule formation when 17β-estradiol was added to the cell-culture medium (31).

Cell Source: Mesenchymal dental stem cells (MDSCs) 

To date, several types of adult stem cells have been isolated from teeth, including dental pulp stem cells (DPSCs) (32), stem cells from human exfoliated deciduous teeth (SHEDs) (33), periodontal ligament stem cells (PDLSCs) (34), dental follicle progenitor stem cells (DFPCs) (35), and stem cells from apical papilla (SCAPs) (36).

**Figure 2:**
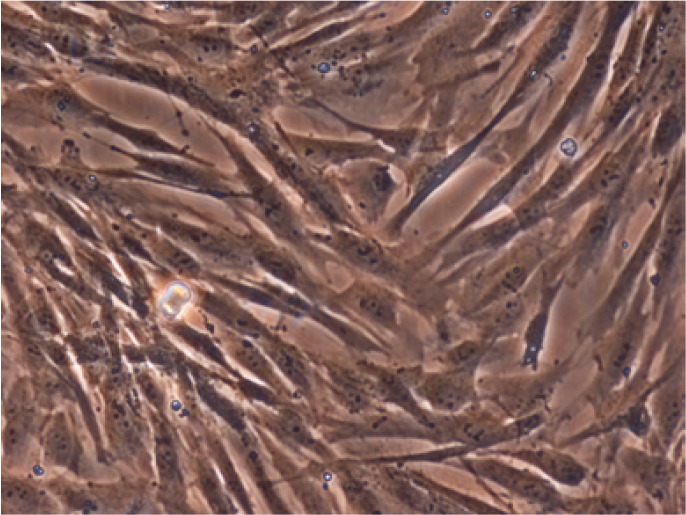
Spindle shaped dental stem cells in culture media. Phase contrast microscopy, original magnification: X100.

MDSCs are multipotent cells that proliferate extensively (maintained for at least 25 passages), can be safely cryopreserved, pos-sess immunosuppressive properties, and express mesenchymal markers. MSDSCs can be isolated using explant cultures or enzy-matic digestion. In addition, the stem cells derived from teeth are large spindle-shaped cells with a large central nucleus abundant cytoplasm, and cytoplasmic extensions in culture (Fig. 2). These adherent cells are morphologically identical to the mesenchymal stem cells obtained from bone marrow (BMMSCs) (32). MDSCs can differentiate in vitro into cells of all of the germinal layers, including ectoderm (neural cells), mesoderm (myocytes, osteo-blasts, chondrocytes, adipocytes, and cardiomyocytes), and endo-derm (hepatic cells) (37).

In 2000, Gronthos et al. (32) discovered a new type of stem cells from adult human dental pulp that have the ability to differentiate into odontoblasts/osteoblasts, adypocites and neural cells. These were termed dental pulp stem cells (DPSC) (32). Transplanted ex vivo expanted DPSC mixed with hydroxyapatite/ tricalcium phosphate form ectopic dentin/pulp-like complexes in immunocompromised mice (32). These polls of heterogeneous DPSC form vascularizad pulp like tissue and are surrounded by a layer of odontoblast-like cells expressing factors that produce dentin containing tubules similar those found in natural dentin (32). In addition, DPSCs express mesenchymal markers as CD73, CD90 and CD105 (37). Stem cells from human exfoliated deciduous teeth (SHEDs), also termed immature, are MDSCs from dental pulp tissue of human deciduous teeth with the capacity to differentiate into osteogenic and odontogenic cells, adipocytes, and neural cells (32). As neural crest cell-associated postnatal stem cells, SHED express a variety of neural cell markers including nestin, beta III tubulin, GAD, NeuN, GFAP, NFM, and CNPase (33). Also, SHED are able to form bone when transplanted in vivo and may be an appropriate stem cell resource for treating immune disorders via improved immunomodulatory properties (33). Periodontal ligament stem cells (PDLSCs) isolated from human periodontal ligament also express mesenchymal markers. In vitro, PDLSCs have the ability to differentiate in vitro into adipogenic, osteogenic and chondrogenic cells (34). PDLSCs represent a novel stem cell population in terms of in vivo capacity to differentiate into cells similar to cementoblasts and collagen-forming cells, as evidenced positively en preclinical studies (34). Dental follicle progenitor stem cells (DFPCs) obtained from a mesenchymal tissue that surrounds the developing tooth germ are multipotent stem cells that have immunomodulatory properties, high proliferation potential and capacity to differentiate into odontoblasts, cementoblasts, osteoblasts and other cells implicated in the teeth (35). Furthemore, are able to re-create a new periodontal ligament (PDL) after in vivo implantation (37). Finally, the stem cells from apical papilla (SCAPs) isolated from a precursor of radicular pulp, express mesenchymal markers and can differentiate into odontoblast-like cells and produce dentin-like tissue in both in vitro and in vivo study systems (36). SCAP together with PDLSCs are able to form a root-like structure when seeded onto the hydroxyapatite-based scaffold and implanted in pig jaws (37).

Scaffold

Actually, investigators search the “ideal scaffold” to facilitate the growth, integration and differentiation of stem cells. The scaffold should be biocompatible, non-toxic and have optimal physical features and mechanical properties. Experiments with cell-free scaffolds are especially attractive because of an easier handling process that eliminates the issues associated with the use of stem cells and their expansion in vitro, with storage and shelf-life, cost aspects, immunoresponse of the host and transmission of diseases (38). However there are some disvantages in this method: first, the cells may have low survival rates. Second, the cells might migrate to different locations within the body, possibly leading to aberrant patterns to mineralization. A solution for this problem may be to apply the cells together with a scaffold. This would help to position and maintain cell localization (39).

Many materials have been designed and constructed for tissue engineering approaches, namely natural and synthetic polymers or inorganic materials and composites, which have been fabricated into porous scaffolds, nanofibrous materials, microparticles and hydrogels. Natural materials include collagen, elastin, fibrin, alginate, silk, glycosaminoglycans such as hyaluronan, and chitosan (40). They offer a high degree of structural strength, are compatible with cells and tissues and biodegradable, but are often difficult to process and afflicted with the risk of transmitting animal-associated pathogens or provoking an immunoresponse. Synthetic polymers as poly lactic acid (PLA), poly glycolic acid (PGA), and their copolymer, poly lactic-co-glycolic acid (PLGA) provide excellent chemical and mechanical properties and allow high control over the physicochemical characteristics, such as molecular weight, configuration of polymer chains, or the presence of functional groups. Recently, hydrogels have been explored for tissue engineering applications in more detail. Hydrogels offer numerous interesting properties including high biocompatibility, a tissue-like water content and mechanical characteristics similar to those of native tissue (40).

## Conclusions

Mesenchymal Dental Stem cells derived from teeth are easily accessible multipotent cells with the capacity to differentiate into distinct cell types. This new source of stem cells could be of benefit in cellular therapy and the eventual development of techniques for use in regenerative dentistry and degenerative diseases. Future studies will probably focus on subpopulations of stem cells derived from teeth characterized by specific surface markers, their distinctive properties and their use in clinical applications.
